# MITIGATING SOCIAL ISOLATION AND LONELINESS AMONG OLDER ADULTS

**DOI:** 10.1093/geroni/igae098.0670

**Published:** 2024-12-31

**Authors:** W Quin Yow

**Affiliations:** Chair

## Abstract

Approximately 24% of older adults in the US experience social isolation, a condition linked to numerous physical and mental health issues, including stroke and cognitive decline. However, there is a lack of research in the assessment and intervention of social isolation and loneliness within older populations at the community level. This symposium seeks to address this gap by examining current research progress in assessment and intervention strategies for combating social isolation and loneliness among older adults. Paper 1 explores factors influencing healthcare providers’ comfort levels in screening for social isolation. The author found that important factors include effective communication, interaction, and minority status. Paper 2 evaluates the community-based project “Waigaya project” in suburban Japan aimed at fostering connections among older residents. The project shows effectiveness in addressing social isolation and loneliness among suburban residents. Paper 3 presents survey findings on the impact of online connections in alleviating loneliness among older adults, emphasizing the importance of fostering quality interactions within digital communities. Finally, Paper 4 explores the use of AI-driven conversation agents to encourage social engagement among older adults to combat social isolation and loneliness. They will also present findings from a preliminary study on conversation topics among older adult friends, guiding the chatbot development. The symposium aims to enhance participants’ understanding of two key areas: factors to consider and strategies for implementing community-level screening and interventions targeting social isolation and loneliness among older adults, and the feasibility of leveraging technology to address social isolation and loneliness in older adults.

